# A comparison of pin site complications between large and small pin diameters in robotic-assisted total knee arthroplasty

**DOI:** 10.1186/s40634-023-00584-1

**Published:** 2023-03-10

**Authors:** Sohil S. Desai, Jennifer A. Kunes, Michael B. Held, Mark Ren, Alirio J. deMeireles, Jeffrey A. Geller, Roshan P. Shah, H. John Cooper

**Affiliations:** Department of Orthopedic Surgery, Columbia University Medical Center, 622 W 168th St, PH 11, NY 10032 New York, USA

**Keywords:** Arthroplasty, Replacement, Knee, Robot assisted surgery, Complications, Infection

## Abstract

**Purpose:**

Robotic-assisted total knee arthroplasty typically necessitates use of tracking pins, which can vary in diameter. Complications such as infections and fractures at the pin-site have been observed, but clarification of the effect of pin diameter on complication is needed. The aim of this study is to compare the pin-related complication rates following robotic-assisted total knee arthroplasty between 4.5 mm and 3.2 mm diameter pins.

**Methods:**

In this retrospective cohort study, 90-day pin-site complication rates after robotic-assisted total knee arthroplasty were compared between 4.5 mm diameter and 3.2 mm diameter groups. In total, 367 patients were included: 177 with large pin diameter and 190 with small pin diameter. All four pin sites were evaluated using postoperative radiographs. Cases without orthogonal views or visualization of all four pin tracts were noted. Multivariate logistic regression was used to control for age, which differed between the two cohorts.

**Results:**

The rate of pin-site complications was 5.6% in the large pin diameter cohort and 2.6% in the small pin diameter cohort, with no statistically significant difference between the groups. The adjusted odds ratio for complications in small compared to large diameter group was 0.48, with a *p*-value of 0.18. The most common pin-site complication was infection/persistent drainage, found in 1.9% of patients, followed by intraoperative fracture of the second cortex in 1.4%. Intraoperative fracture could not be ruled out in 96 cases due to inadequate radiographic visualization of all pin sites. There was one postoperative pin-site fracture in the large diameter cohort, which required operative fixation.

**Conclusion:**

This study demonstrates no statistically significant difference in pin-site complication rates after robotic-assisted total knee arthroplasty between 4.5 mm and 3.2 mm pin diameter cohorts, although there was a trend towards increased intraoperative and postoperative pin-site fractures in the 4.5 mm group.

## Introduction

The use of robotic-assisted (RA) total knee arthroplasty (TKA) procedures has rapidly increased in recent years, with the goal of improving limb alignment, component positioning, and soft-tissue balancing [8]. RA devices allow the use of either preoperative imaging or intra-operative stereotactic mapping of osseous landmarks (imageless navigation) to help determine precise femoral and tibial cuts [14]. Moreover, intra-operatively a robotic arm or a handheld cutting device can assist in the actual performance of the procedure.

Precise and accurate osseous resection and component positioning is critical in order to effectively restore the mechanical axis of the lower extremity or achieve the desired kinematic alignment [14]. Postoperative varus malalignment has been shown to increase the amount of tibial component migration and decrease the long-term survival rates of TKA implants [10, 20]. A driving force in the recent increase in popularity of RA-TKA is the ability for these technologies to improve accuracy and precision in placing components in the desired position [1, 5, 8, 11, 19, 20].

However, the addition of any new technology may introduce new problems. In order to fix the navigation trackers to the distal femur and tibia, a total of four temporary navigation pins must be placed. While the placement of pins is generally a benign procedure, there remains a risk for pin-site fracture, pin-site infections, wound complications, neurovascular injury, and pin breakage in patients undergoing RA-TKA. The reported rate of pin-site complications following computer navigated and RA-TKA has ranged between 0.3% to 1.9% [7, 13, 17, 18]. Interestingly, not all manufacturers of these systems use the same diameter pins, and it has been suggested that the use of larger diameter pins can increase the risk of pin-site fracture [2, 16].

The purpose of this study was to compare pin-related complication rates following RA-TKA between 4.5 mm and 3.2 mm diameter pins during a 90-day follow up period. We hypothesized that the large pin diameter system would result in an increased rate of pin-site complications.

## Methods

### Data collection & analysis

Institutional Review Board (IRB) approval was obtained through Columbia University before study initiation (#AAAT8150). This retrospective cohort study compared the 90-day pin-site complication rate between 177 patients with the large pin diameter (LPD, 4.5 mm) system (Navio, Smith and Nephew, London, UK) and 190 patients with the small pin diameter (SPD, 3.2 mm) system (ROSA, Zimmer Biomet, Warsaw, Indiana). The study included consecutive patients at a single large academic institution undergoing primary robotic-assisted arthroplasty between January 2017 and August 2021. Patients of three senior attending surgeons who used both technologies were included in this study. Choice of RA-TKA system was based on surgeon preference. Revision arthroplasties, arthroplasties that did not utilize either the 4.5 mm or 3.2 mm pin diameter systems detailed above, and patients without 90-days of postoperative follow-up were excluded. The electronic medical record was retrospectively reviewed for demographic data (gender, age at surgery, body mass index [BMI]) and perioperative data (American Society of Anesthesiologists [ASA] scores, laterality, total non-pin-site complications, reoperation rate). Each of these demographic and perioperative data, including total non-pinsite complications (i.e., superficial infection, prosthetic joint infection, wound dehiscence, periprosthetic fracture, arthrofibrosis), were evaluated as potential confounders in the comparison of the primary outcome of total pin-site complications. When pin-site complications occurred, the type (intraoperative fracture, postoperative fracture, infection/drainage, delayed wound healing, neurovascular injury, pin or drill bit breakage), location (femoral versus tibial), and time to complication were recorded. Pin-site infection/drainage and delayed wound healing included only tibial pin sites, as femoral pin sites were placed through the primary incision. Pin-site infection/drainage included patients with persistent local drainage or erythema necessitating the use of post-operative antibiotics. To evaluate for intraoperative pin-site fractures, immediate postoperative radiographs taken in the post anesthesia care unit (PACU) were reviewed. Some cases were recorded as “inconclusive” if one or more of the four pin tracts was not visualized on the radiograph (i.e., image was not distal enough to capture distal tibial pin), or if orthogonal views were unavailable. Radiographs were each independently reviewed by two authors (S.S.D, J.A.G.).

Continuous variables were recorded as mean and standard deviation unless otherwise indicated and compared with students t-test. Categorical variables were recorded as count and percentage and examined with logistic regression. Outcomes were analyzed per patient with multivariate logistic regression, controlling for demographic and perioperative parameters that differed between the groups. Significance is set to *p* < 0.05.

### Study Patients

Overall, 367 patients were included (177 LPD, 190 SPD) in this study. There was no difference in sex, BMI, ASA level, or laterality between the cohorts (Table 1). Age at surgery was significantly lower in the SPD group compared to the LPD group (68.1 years versus 71.4 years, respectively, *p* < 0.01). There was no significant difference in overall rates of non-pin-site complications between the LPD and SPD systems (10.2%, 7.4%, respectively, *p* = 0.29), or in overall reoperation rate (4.0%, 3.2%, respectively, *p* = 0.63) (Table 2).

**Table 1:** Demographic characteristics of included patients

		LPD (*n* = 177)	SPD (*n* = 190)	*p*-value
**Sex**, n (%)	**Male**	52 (29.4)	72 (37.9)	.11
	**Female**	125 (70.6)	118 (62.1)	
**Age**, years		71.4 ± 9.5	68.1 ± 10.2	< 0.01
**BMI**		30.2 ± 5.8	30.4 ± 5.7	.69
**ASA**		2.34 ± 0.50	2.43 ± 0.58	.12
**Laterality**, n (%)	**Right**	92 (52.0)	99 (52.1)	1
	**Left**	85 (48.0)	91 (47.9)	

*LPD* large pin diameter robotic-assisted total knee arthroplasty system, *SPD* small pin diameter robotic-assisted total knee arthroplasty system

**Table 2:** Complications by Type

	All (*n* = 367)	LPD (*n* = 177)	SPD (*n* = 190)	Adjusted OR^a^	*p*-value^a^
**Total Pin-Site Complications**, n (%)	15 (4.1)	10 (5.6)	5 (2.6)	0.48 (0.16, 1.44)	.18
Intraoperative pin-site fracture	5 (1.4)	4 (2.8)	1 (0.5)	0.24 (0.03, 2.21)	.16
Postoperative pin-site fracture	1 (0.3)	1 (0.6)	0 (0.0)	-	.25
Pin-site infection/drainage*	7 (1.9)	4 (2.2)	3 (1.6)	0.69 (0.15, 3.19)	.63
Pin-site delayed wound healing*	2 (0.5)	1 (0.6)	1 (0.5)	0.93 (0.06, 15.0)	.89
Neurovascular injury	0 (0.0)	0 (0.0)	0 (0.0)	-	-
Pin or drill bit breakage	0 (0.0)	0 (0.0)	0 (0.0)	-	-
**Non-Pin-Site Complications**, n (%)	32 (8.7)	18 (10.2)	14 (7.4)	0.67 (0.32, 1.41)	.29
**Reoperation**, n (%)	13 (3.5)	7 (4.0)	6 (3.2)	0.76 (0.25, 2.34)	.63

*LPD* large pin diameter robotic-assisted total knee arthroplasty system, *SPD* small pin diameter robotic-assisted total knee arthroplasty system

^*^Pin-site infection/drainage and delayed wound healing included only tibial pin sites, as femoral pin sites were placed through the primary incision

^a^Odds ratio and *p*-value adjusted for age via logistic regression

### Technical details of pin diameter and placement

**Fig. 1:**
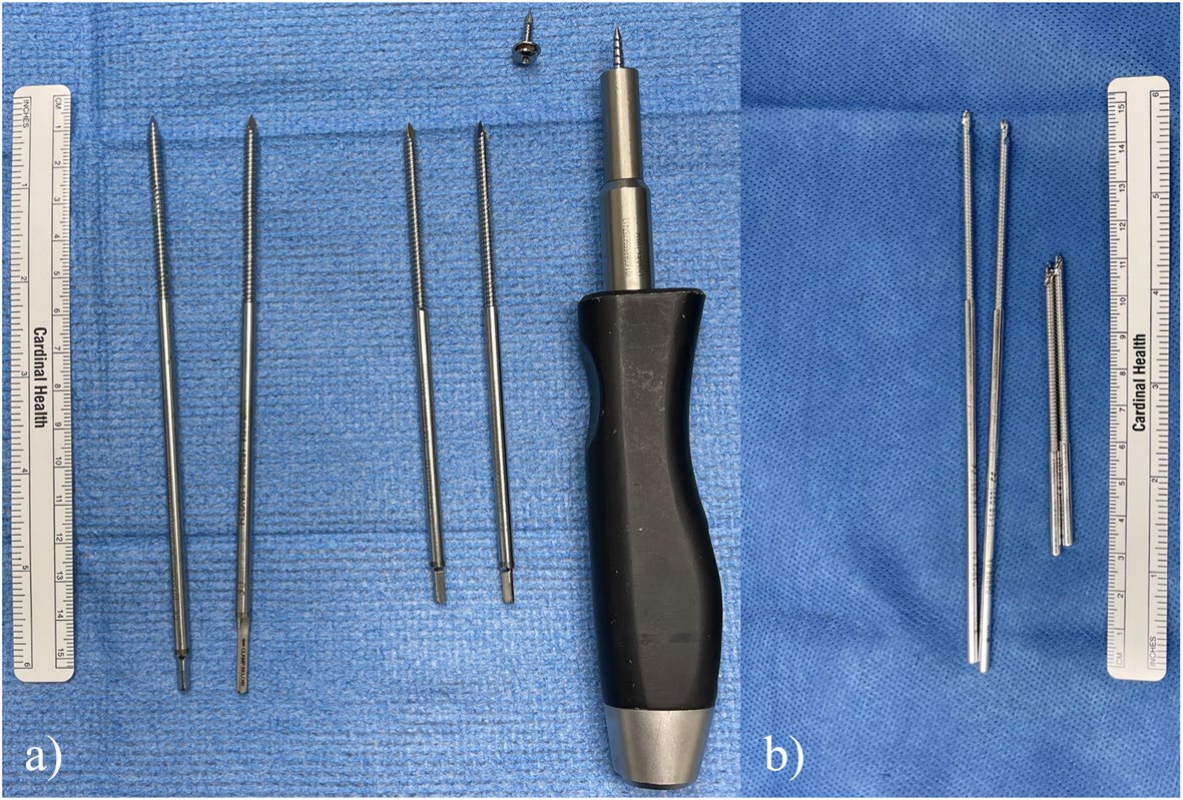
Tracking pins from the **a** large pin diameter and **b** small pin diameter robotic-assisted total knee arthroplasty systems. The longer pins in both images are the femoral pins, while the shorter pins are the tibial pins

Each RA-TKA in this analysis was performed with a midline incision followed by a standard medial parapatellar or midvastus approach and exposure. Four temporary half pins – two in the distal femur and two in the proximal tibia – were placed with power drill in order to attach the optical tracking arrays and enable robotic assistance in both groups. The femoral tracking pins for the LPD system were 4.5 mm in diameter and 150 mm in length, while the tibial pins were 4.5 mm in diameter and 120 mm in length. As for the SPD system, the femoral tracking pins were 3.2 mm in diameter and 150 mm in length, while the tibial pins were 3.2 mm in diameter and 80 mm in length (Fig. 1). There are no additional technical differences in the placement of the pins between the LPD and SPD systems.

In both groups, the two distal femoral pins were placed through the primary incision in the metadiaphyseal region, placed anterior to posterior. The proximal tibial pins were placed 4 finger breadths distal to the tibial tubercle into the tibial crest, through a longitudinal stab incision. The second pin was placed a fixed distance distally using a tissue protector. All pins were placed bicortically, stopping at the pitch change heard as the threads engage the far cortex. Regarding pin design, all pins are fluted, half-threaded, and self-cutting.

At the conclusion of the procedure the tibial pin site incisions were closed with absorbable monofilament buried sutures. This was then sterilely dressed with a polyester mesh with liquid adhesive.

## Results

### Pin-site complications

**Fig. 2:**
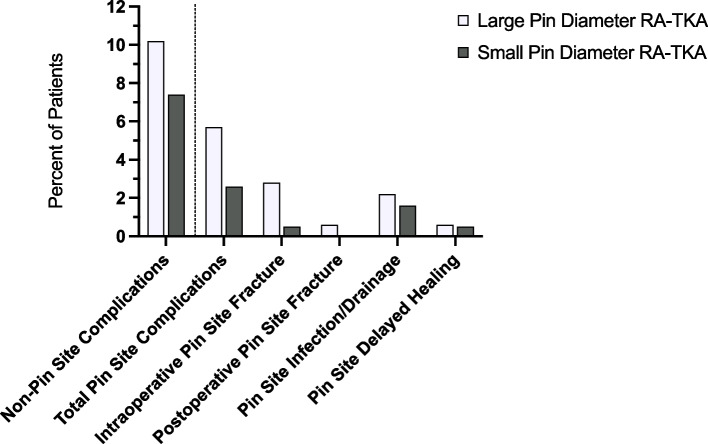
Complications by pin diameter group (RA-TKA = robotic-assisted total knee arthroplasty)

**Fig. 3:**
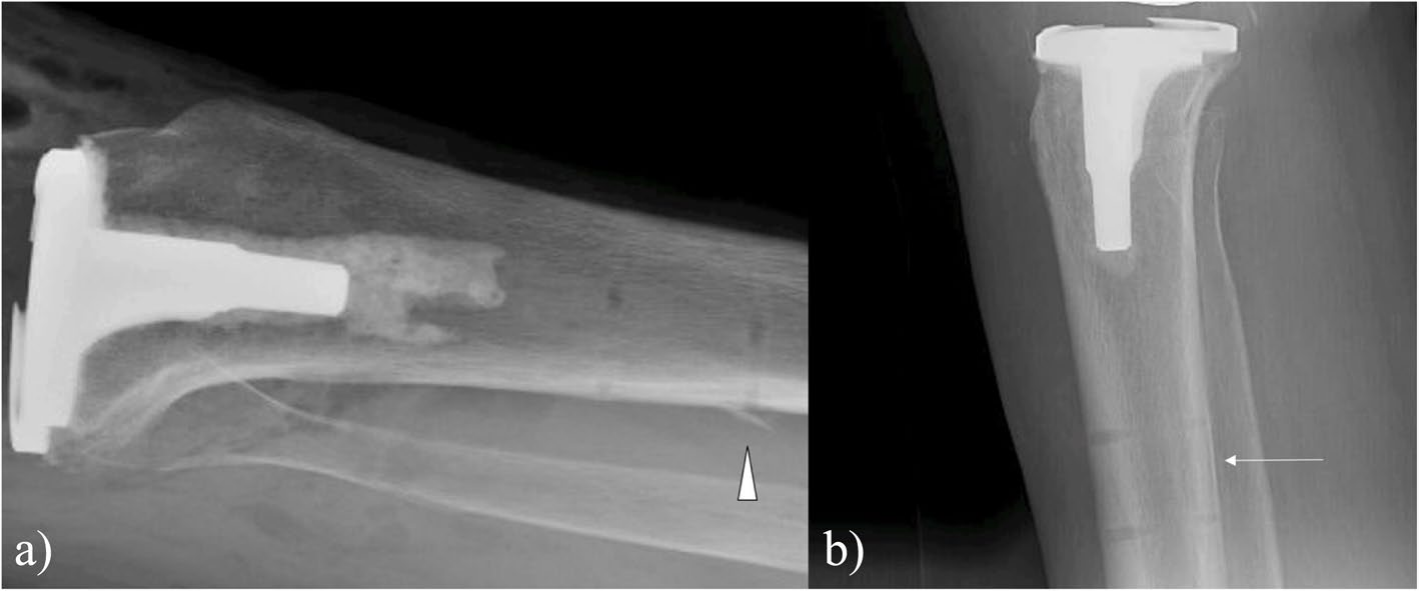
**a** Lateral radiograph of a 70-year-old female with an intraoperative pin site cortical fleck (arrowhead) involving the distal tibial pin site of her right knee. **b** Lateral radiograph of an 83-year-old female with an intraoperative nondisplaced pin-site fracture of her right tibia as evidence by linear lucency (arrow) noted along the posterior aspect of the proximal tibial shaft between both pin sites. Both cases involved the large pin diameter robotic-assisted total knee arthroplasty system

**Fig. 4:**
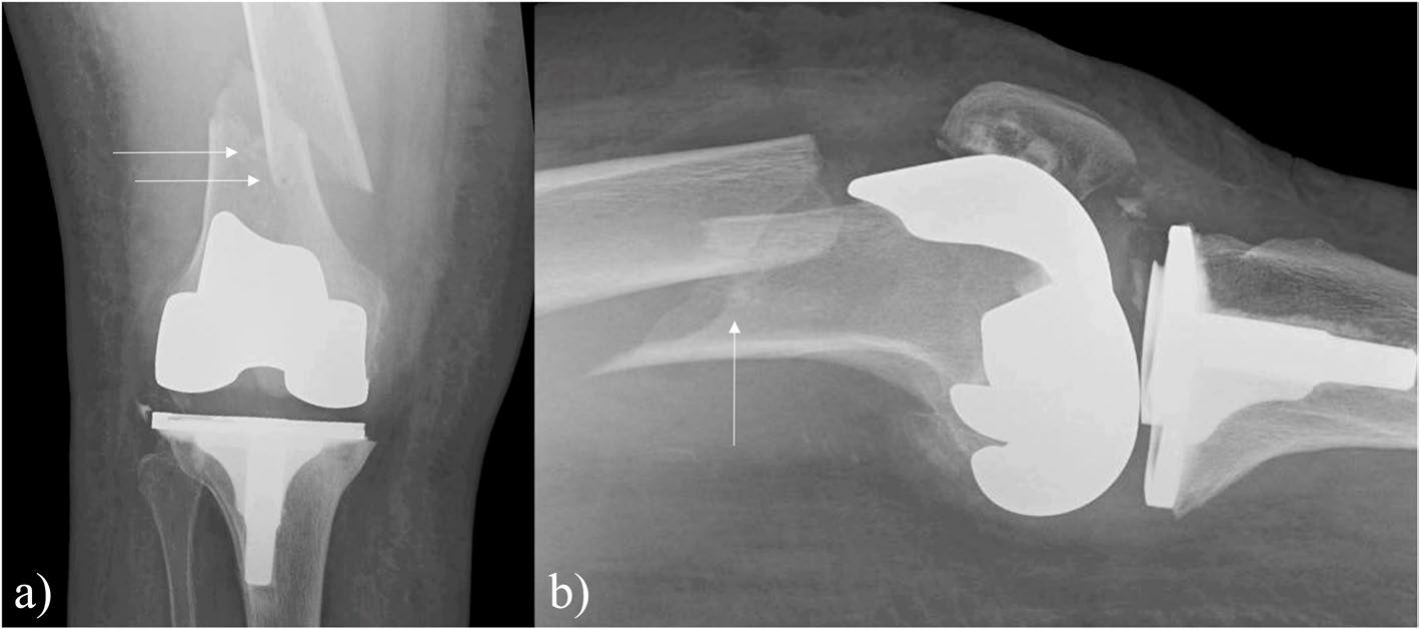
**a** AP and **b** lateral radiographs of a right distal femur postoperative pin-site periprosthetic fracture in a 75-year-old female following robotic-assisted total knee arthroplasty with the large pin diameter system. Arrows demarcate pin tracts, with the fracture line seen passing through the proximal pin tract

The overall rate of pin-site complications was 4.1% (15 out of 367 cases), and there was no statistically significant difference between the LPD and SPD cohorts (5.6%, 2.6%, respectively, *p* = 0.18) (Table 2). A post hoc power analysis revealed that 1,314 patients would be required to identify a significant difference with the standard effect size (β = 0.20, α = 0.05). Odds ratios and *p*-values were adjusted for age via logistic regression. With reference to the LPD group, the adjusted odds ratio (aOR) for pin-site complications in the SPD group was 0.48 (95% CI 0.16 to 1.44), but this finding was not statistically significant (*p* = 0.18). The most common pin-site complication was infection/persistent drainage (2.2% LPD, 1.6% SPD, aOR = 0.69, *p* = 0.63) (Fig. 2). Intraoperative pin-site fracture was the next most common complication, with 4 identified in the LPD group and 1 identified in the SPD group (2.8%, 0.5%, aOR = 0.24, *p* = 0.16) (Fig. 3). There were 96 inconclusive radiographs within the analysis (*n* = 59 LPD, *n* = 37 SPD, 26.2% overall), in which there was no obvious intraoperative fracture identified, but fracture could not be ruled out due to the aforementioned reasons. Removal of these patients from analysis for intraoperative pin-site fracture still demonstrates no significant difference in rate of fracture between the two groups (*p* = 0.10). One postoperative pin-site fracture was identified, occurring within the LPD group (*p* = 0.25) (Fig. 4).

Thirteen of the pin-site complications involved the tibial pin sites (all 5 intraoperative pin-site fractures, all 7 pin-site infection/drainage, 1 delayed wound healing), 1 involved the femur (postoperative pin-site fracture), and 1 was unspecified (delayed wound healing). Most pin-site complications were either intraoperative or presented within one month postoperatively (Table 3). Age at surgery, laterality, BMI, and ASA level were not significantly associated with increased risk of pin-site complications or fracture specifically. There was no significant difference in pin-site complication rate by surgeon.

**Table 3:** Time to Pin-Site complication

Time to Complication	n
Intraoperative	5
0 to 1 month	6
1 to 2 months	1
2 to 3 months	2

^*^1 complication unspecified

There were 5 cases of periprosthetic joint infection (PJI) each in LPD and SPD groups (2.8% LPD, 2.6% SPD). The rate of PJI did not significantly differ between groups ( = 0.910). The rate of all superficial wound infection, including pin-site and non-pin-site, was 5.1% in the LPD group and 2.1% in the SPD group (*p* = 0.123).

## Discussion

The aim of this study was to compare the rate of pin-site complications between two RA-TKA systems with different diameter tracking pins. While prior studies have suggested that larger pin diameters may increase risk for pin-site complications, this is the first study to directly compare the rate of complications between different sized pins [16]. In the present analysis, there was a 4.1% overall rate of pin-site complications. There was a lower rate of pin-site complications in the SPD cohort compared to the LPD cohort, but this difference was not statistically significant (2.6%, 5.6%, aOR = 0.48, respectively, *p* = 0.18). The most common complication was pin-site drainage/infection (1.9% overall incidence), of which there were 4 (2.2%) in the LPD group and 3 (1.6%) in the SPD group, with no statistically significant difference between the groups (aOR = 0.69, *p* = 0.63). All cases of local pin-site infection resolved at the pin-site uneventfully with antibiotic treatment.

The addition of pin placement to total joint arthroplasty introduces the potential for several complications, including pin-site infection, delayed wound healing at pin sites, damage to neurovascular structures, breakage of pins, and pin-site fracture. Thus far, the current literature has shown these complications are relatively rare. The overall rate of pin-site complications following computer navigated and RA-TKA has ranged between 0.3% to 1.9% [7, 9, 12, 13, 17]. Pin-site infection is the most commonly reported complication, with rates ranging between 0.3% to 1.2% [7, 12, 13, 17, 18]. In the present study, the incidence of pin-site infection/drainage was slightly higher than previous reports of pin-site infection, which may have been related in part to our broad definition of this complication (i.e., cases of pin-site drainage that may have had no associated infection were still included in the group). Avoiding transcortical and juxtacortical drilling has been suggested to decrease the risk of infection [6]. Some authors argue that pin-site infections and wound healing complications can be nearly avoided altogether when pinning is performed through the operative incision [3, 7]. Both RA-TKA systems in the present analysis involved percutaneous placement of the tibial pins.

Pin sites introduce stress risers in the bone, and although rare, postoperative pin-site periprosthetic fracture can be a serious complication. Pin placement can also cause intraoperative fracture, more frequently occurring in the far cortex as the pin is tightened into place. In one systematic review, the incidence of postoperative pin-site periprosthetic fracture in RA-TKA was 0.16%; no intraoperative fractures were reported [3]. In a more recent systematic review, 13 postoperative pin-site fractures and 0 intraoperative pin-site fractures were reported out of the 4,641 RA-TKAs (0.28%, 0.0%, respectively) analyzed from non-case report studies [18]. Case reports were discussed separately in this review, with 21 reports of postoperative pin-site fracture and only 1 report of intraoperative pin-site fracture [18]. Notably, both pin types used in this study were self-drilling, self-tapping designs. Prior studies examining pin tip design on complications are sparse, but the available literature supports the use of self-drilling self-tapping pins, suggesting decreased heat necrosis as an explanation for improved clinical outcomes [16].

The use of larger diameter tracking pins has been suggested to increase the risk of fracture [2]. Indeed, the 1 postoperative periprosthetic pin-site fracture in our analysis occurred within the LPD group, although this did not represent a statistically significant difference in incidence (*p* = 0.25). This displaced femur fracture, which passed through the proximal femoral pin tract, required open reduction and internal fixation (Fig. 4). Five intraoperative pin-site fractures were identified in this analysis, with 4 occurring in the LPD group and 1 occurring in the SPD group (1.4% overall, 2.8% LPD group, 0.5% SPD group, aOR = 0.24, *p* = 0.16). Of note, these intraoperative fractures were either minor cortical “flecks” or posterior cortex lucencies between the two pin sites, which in some cases were audibly identifiable by the surgeon during final hand-tightening of the pin (Fig. 3). None of these intraoperative fractures were symptomatic or required modification of the normal postoperative weight-bearing protocol. This is in contrast to the postoperative fracture in our analysis, and several of the postoperative pin-site fractures identified in Thomas and colleagues’ systematic review, which required modified weight-bearing and operative intervention [18].

All “inconclusive” cases had no overt intraoperative fracture in all visualized pin sites, but fracture could not be ruled out because one or more pin site was not visualized, or orthogonal views were not available. The high overall rate of “inconclusive” cases (*n* = 96, 26.2%), as well as the clinical irrelevance of each of our 5 minor cortical fractures, somewhat complicates the reporting of intraoperative pin-site fractures. Nevertheless, this appears to be the first analysis to discuss the prevalence of these intraoperative fractures, which as mentioned previously were frequently accompanied by an audible “crack” during tightening of the pin. Clinicians should continue to be aware of this possible complication, as displaced fractures can occur and require appropriate management [18].

Intraoperative complications also include breakage of drill bits or pins, as well as malalignment. These complications are reported to be rare (0.3% drill bit breakage, 0.1% malalignment) and have not been reported to adversely affect outcomes [7]. Neither occurred in any of the cases in this analysis. Hardware breakage can occur with placement of the reference pins too close to the femoral or tibial bone cuts. With increased proximity to the joint, the pins are at risk for breakage from the cutting tools [7]. Thomas et al. found that pin breakage occurred in 0.5% of cases, but this did not lead to RA abandonment or change of clinical outcome in any of the cases [18]. Finally, neurovascular damage is another rarely reported complication. Gulhane et al. describe the only reported case of vascular injury, involving bleeding from a branch of the superficial femoral artery that required operative hematoma evacuation and tamponade [4]. There was no neurovascular injury in this analysis. Interestingly, 13 of the 14 total pin-site complications in this study with a specified location involved a tibial pin site. The increased rate of tibial pin-site complications may be associated with the use of an additional stab incision for placement of tibial pins, while femoral pins can be placed through the primary operative incision [7]. It is unclear why intraoperative pin-site fractures in our analysis occurred only in the tibia.

This study is not without limitation. We were able to radiographically identify 5 intraoperative pin-site fractures among our 367 cases, but we believe this underestimates the true number intraoperative fractures that occurred for two reasons. Firstly, there was a high rate of inconclusive PACU radiographs (*n* = 96, 26.2%) encountered during our evaluation for intraoperative pin-site fractures. The lack of orthogonal views or inclusion of all 4 pin sites in these cases prevented our ability to completely exclude fracture. Secondly, it is possible that some intraoperative fractures occurred but were not identifiable radiographically, even if appropriate radiographs were available, due to the minimally displaced nature of these fractures (Fig. 3b). Additional limitations also existed. Due to the relatively low overall number of complications, we may have been underpowered to identify a difference between the two groups. An a priori power analysis was not performed prior to data collection. Additionally, the retrospective nature of this study introduces the potential for bias and confounding as subjects were not randomly allocated into each cohort. We adjusted for age, which could increase risk for pin-site fracture and statistically significantly differed between the two groups [15]. It is possible that other potential confounders existed. Finally, this study may have been strengthened by expanding follow up to 180 days, as it is possible that postoperative pin-site periprosthetic fractures and other complications occurred after the 90-day study period.

## Conclusion

Pin-site complications are uncommon following robotic-assisted total knee arthroplasty but should continue to be a consideration during surgical management and follow up. There was no statistically significant difference in complication rates demonstrated in this analysis (5.6% in the 4.5 mm pin diameter group versus 2.6% in the 3.2 mm pin diameter group, *p* = 0.36). However, there was a trend towards increased intraoperative pin-site fracture (2.8% in the 4.5 mm diameter group versus 0.5% in the 3.2 mm diameter group, *p* = 0.18) and postoperative pin-site fracture (0.6% in 4.5 mm group, 0.0% in 3.2 mm group, *p* = 0.25) in the large pin diameter group.

## Data Availability

Data can be made available upon direct request to the corresponding author.
